# Comparison of Serum Concentrations of Ustekinumab Obtained by Three Commercial Assays in Patients with Crohn’s Disease

**DOI:** 10.1093/jcag/gwaa003

**Published:** 2020-03-31

**Authors:** Christine Verdon, Niels Vande Casteele, Valérie Heron, Pascale Germain, Waqqas Afif

**Affiliations:** 1 McGill University Health Centre, McGill University, Montreal, Quebec, Canada; 2 Department of Medicine, University of California San Diego, La Jolla, California, USA

**Keywords:** Biomarkers, Therapeutic drug monitoring, Ustekinumab

## Abstract

**Background:**

Data on the association of ustekinumab (UST) drug concentrations and clinical outcomes are conflicting. We assessed serum UST drug and anti-UST antibody concentrations using three commercially available assays.

**Methods:**

Sixty-one blood samples were analyzed for serum UST drug and anti-UST antibody concentrations using three assays: one homogeneous mobility shift assay (HMSA, Prometheus, Assay A), and two enzyme-linked immunosorbent assays (ELISA; Progenika, Dynacare, Assay B and Theradiag, Assay C).

**Results:**

The median (IQR) serum UST concentrations for the three assays were: Assay A 7.50 (5.35 to 12.88) µg/mL, Assay B 4.02 (2.46 to 6.95) µg/mL and Assay C 4.35 (2.62 to 7.50) µg/mL. A Kruskal–Wallis test confirmed a statistically significant difference between the different assays, X^2^(2) = 30.606, *p* < 0.001. Linear regression showed near twofold increased difference in the absolute drug concentrations between the HMSA and either ELISA. Linear quantitative correlation was observed for all three assays (*r* = 0.836 for A versus B, *r* = 0.792 for A versus C, *r* = 0.936 for B versus C; *p* < 0.01). The intraclass correlation coefficient (ICC) between assay A and B was 0.649 (95% confidence interval [CI] −0.208 to 0.874); assay A and C was 0.671 (95% CI −0.165 to 0.878); and assay B and C was 0.958 (95% CI 0.928 to 0.975); *p* < 0.001. No anti-UST antibodies were detected.

**Conclusion:**

A good correlation was observed for serum UST drug concentrations and a good agreement was observed between the ELISA tests. However, agreement was poor between the HMSA and each ELISA tests. Clinical recommendations regarding drug concentrations should be based on assay type used.

## Introduction

Therapeutic drug monitoring (TDM) of biologics, including the measurement of drug and anti-drug antibody concentrations and antibodies, has been shown to improve Inflammatory Bowel Disease (IBD) outcomes with greater drug durability, reduced risk of antibody formation, serious infusion reactions, decreased surgeries and hospitalizations (1). There are clear guidelines (2,3) in regards to the therapeutic drug concentration necessary to achieve mucosal healing (4–6) with anti-tumour necrosis factor-α (TNF) molecules (infliximab, adalimumab, golimumab and certolizumab). In contrast, evidence on TDM for ustekinumab (UST), a monoclonal antibody to the shared p40 subunit of the pro-inflammatory interleukin (IL)-12 and IL-23 cytokines, is scarce. UST has been shown to be effective in inducing and maintaining remission in patients with moderate to severe Crohn’s disease (CD) (7). Moreover, UST is less immunogenic than anti-TNF molecules, with a reported rate of anti-UST antibodies to be 2.3% (4). An exposure–response relationship has nevertheless been observed supporting the concept of TDM assessment with UST (8). However, there are limited and conflicting data available in regards to TDM and serum UST concentrations associated with mucosal healing in IBD patients. Adedokun et al. (9) performed a phase III clinical trial analysis, looking at pharmacokinetics and exposure–response relationship with UST in CD patients and revealed that through levels of >0.8 to 1.4 μg/mL were associated with maintenance of clinical remission in a greater number of patients. They used a validated electrochemiluminescent immunoassay (ECLIA) method on the Meso Scale Discovery (MSD) platform (Gaithersburg, MD) to obtain serum concentrations of UST. However, a small cohort of 19 patients in Dublin showed a serum drug level > 3.6 μg/mL was associated with clinical remission at week 8 (10). Meanwhile, a cohort of 42 patients in Lille, France, showed no association between the drug level and clinical response (11). Both of these studies used an ELISA assay. The latest available study by Battat et al. (12) showed UST levels above 4.5 μg/mL at week 26 were associated with endoscopic response in 75% in a cohort of 62 CD patients. In this latter study, a drug-tolerant liquid phase homogeneous mobility shift assay (Prometheus Laboratories Inc) was used to ascertain UST serum concentrations. One possible reason for the wide range of serum UST concentrations reported could be the different end points of clinical remission and endoscopic improvement used in these studies. Alternatively, it may lie in the fact that different assays were used to measure UST drug concentrations and anti-UST antibodies. Our aim was to perform a comparative evaluation of UST drug concentrations obtained using three different drug testing assays used in Canada, USA and Europe. Assays from Prometheus (drug-tolerant HMSA), Dynacare (ELISA Progenika) and Theradiag (ELISA) were assessed.

## Methods

### Samples

Blood samples from 40 CD patients currently being treated with UST were collected as part of another research study assessing loss of response to UST (8); a total of 61 blood samples were obtained. Once collected, the blood sample were centrifuged, serum was isolated and stored at −8°C until required for testing serum drug concentration. Separate aliquots of each sample were then sent to each assay provider for serum quantification of UST drug concentrations testing and UST anti-drug antibodies, per each providers protocol. No spiked samples or control samples were used.

### UST and Anti-UST Antibody Assays

Three different assays were evaluated. The first assay was a drug-tolerant liquid-phase homogeneous mobility shift assay (HMSA) made by Prometheus Laboratories Inc (San Diego, CA). The lower limit of detection (LLOD) was <0.9 µg/mL. Assay B and C were both enzyme-linked immunosorbent assay (ELISA) made by Progenika (Spain, used by Dynacare) and Theradiag (France), respectively. The LLOD for Dynacare was ≤0.7 µg/mL and for Theradiag, it was <0.04 µg/mL. Modification to the data set was made by adjusting the LLOD value to ½ LOD for a more accurate statistical analysis (i.e., Prometheus LLOD is <0.9 µg/mL with one decimal value, hence data set was adjusted to 0.4 µg/mL; Dynacare LLOD is ≤0.70 µg/mL with 2 decimal values, hence data set was adjusted to 0.35 µg/mL; no results were found to be below the LLOD using Theradiag assay). The higher limit of quantification (HLOQ) was >25 µg/mL for Prometheus, >20 µg/mL for Dynacare and >100 µg/mL for Theradiag.

### Statistical Analysis

Statistical analysis was performed using SPSS statistical software package version 24.0 (IBM, New York, NY). Descriptive (median, interquartile range) and comparative analyses were performed using Spearman test, intraclass correlation coefficient (ICC), linear regression plots and Bland-Altman plots. ICC (absolute agreement) was used to quantify the degree of agreement between assays, and was performed using the two-way mixed single measures test where complete agreement is represented by a value of 1. Bland-Altman plots were created to assess agreement between both assays graphically. In short, the difference between the two measurements was presented on the Y-axis and the average of the two measurements on the X-axis. Ideally, a flat line result confirms agreement between two assays.

## Results

A total of 61 samples from 40 patients with CD were collected and analyzed using each of the three assays. One sample was excluded from the analysis due to results being above the HLOQ for UST in two out of three assays, which would falsely skew data. Therefore, 60 samples were included in the final statistical analysis.

**Figure 1.**
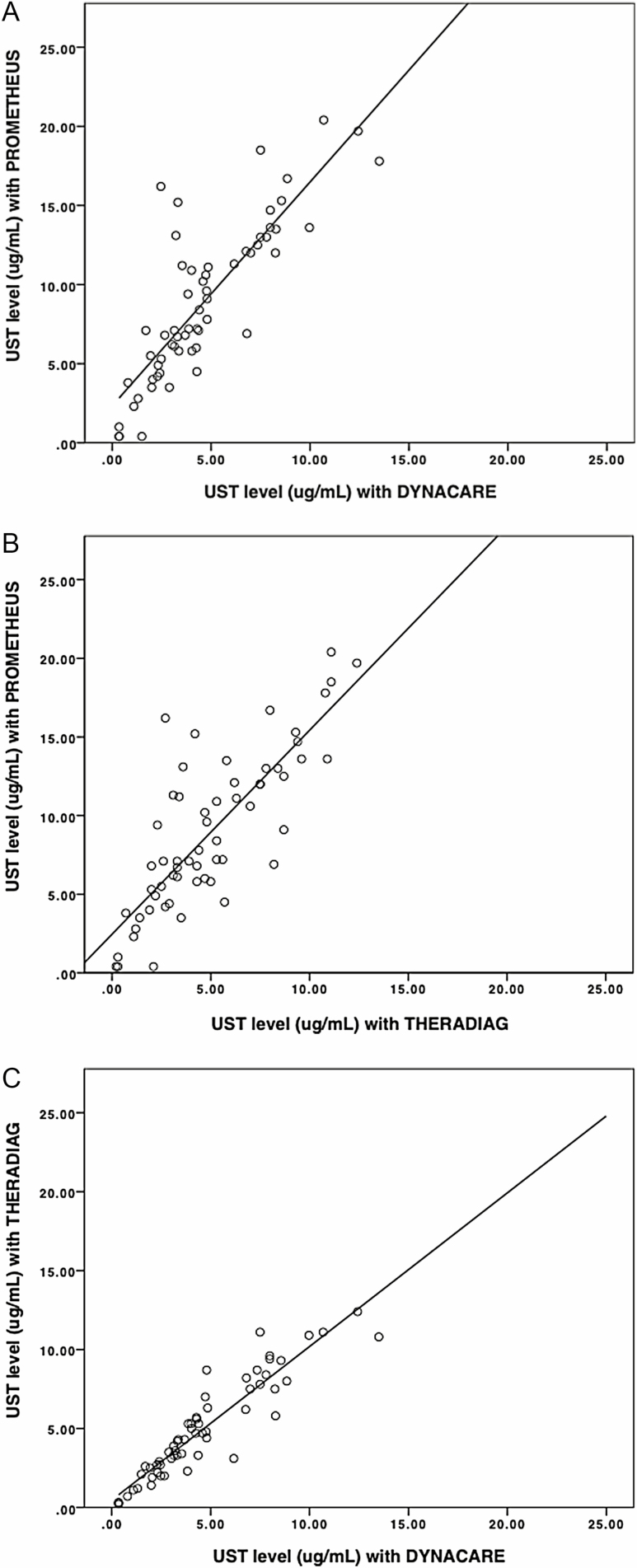
Linear regression plot for UST levels **(a)** Prometheus (Assay A) versus Dynacare (Assay B) **(b)** Prometheus (Assay A) versus Theradiag (Assay C) **(c)** Dynacare (Assay B) versus Theradiag (Assay C).

The median serum UST concentration (interquartile range [IQR]) for the three assays were: Assay A 7.50 (5.35 to 12.88) µg/mL, Assay B 4.02 (2.46 to 6.95) µg/mL and Assay C 4.35 (2.62 to 7.50) µg/mL. The Kruskal–Wallis test confirmed a statistically significant difference in UST concentration between the different assays, X^2^(2) = 30.606, *p* < 0.001. Linear regression plots were performed to show the nearly twofold difference between Assay A and the ELISA assays (Figure 1). Four patients had undetectable drug concentrations on at least one of the assays. There were no anti-UST antibodies detected in any of the samples using any of the assays.

All three UST assays showed a linear quantitative correlation, but the highest correlation was obtained when comparing Assay B and C with a Spearman *r* of 0.936. Comparison of Assay A and B resulted in a Spearman *r* of 0.836, and *r* of 0.792 for Assay A versus C (*p* < 0.01).

**Figure 2.**
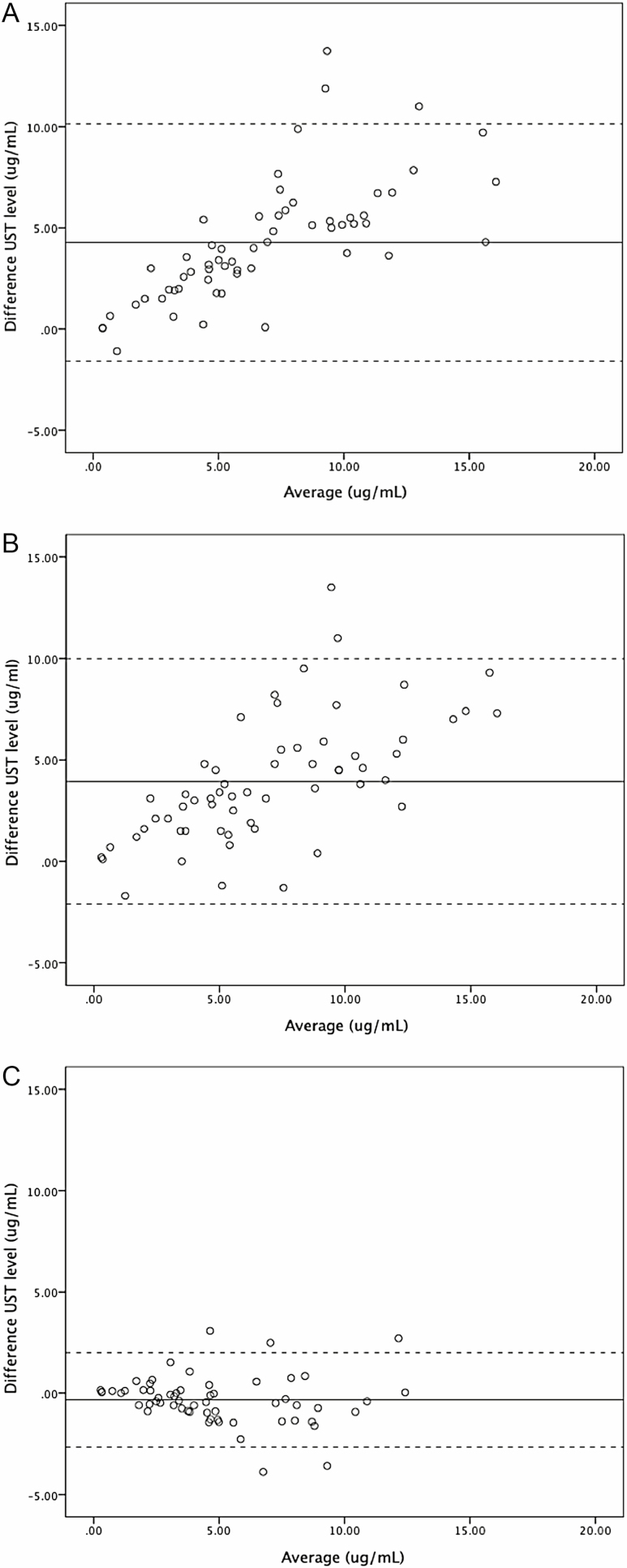
Bland–Altman Plots for UST levels comparing two assays methods. Dotted lines represent the 95% limits of agreement. **(a)** Prometheus (Assay A) versus Dynacare (Assay B); **(b)** Prometheus (Assay A) versus Theradiag (Assay C); **(c)** Dynacare (Assay B) versus Theradiag (Assay C).

The ICC was used to quantify the degree of agreement between the UST levels obtained using two different assays. The best agreement was found between assay B and C with a coefficient of 0.958 (95% confidence interval [CI] 0.928 to 0.975). The Bland–Altman plot of assay B versus C shows the average of the differences is close to zero, indicating the two assays are producing similar results (Figure 2c). However, as the average of the two measurements increases, the difference starts to increase. The agreement coefficient was lower with the other sets of assays: the ICC between assay A and B was 0.649 (95% CI −0.208 to 0.874), and 0.671 (95% CI −0.165 to 0.878) between assay A and C. For the latter sets, the Bland–Altman plots show a more scattered, less linear data set, which moves away from the zero line (Figure 2a and b).

## Discussion

Anti-TNF biologics have revolutionized the treatment of inflammatory bowel disease. However, in the real-life setting about 10 to 30% of IBD patients will be primary non-responders, and between 10 and 25% of patient who do respond initially, will develop secondary loss of response (13). This loss of response is often related to development of drug antibodies. Therapeutic drug monitoring (TDM) has been key in ensuring optimization of anti-TNF therapies (14,15). Moreover, TDM was shown to assist with the decision making of subsequent therapies following anti-TNF drug failure (2,3). New molecules, such as UST, are still being evaluated to determine the adequate drug level range that is associated with clinical and endoscopic remission. UST drug concentrations may also play a significant role in managing patients with loss of response, particularly in guiding clinical decisions around dose escalation, re-induction or discontinuation of drug (8).

Commercially available assays used to determine drug concentration levels must be validated to ensure proper interpretation of the results in a clinical setting. Similar evaluations of assays were performed with infliximab and adalimumab assays by different groups (16–20). Marini et al. (20) performed an evaluation of four ELISA assays for infliximab and demonstrated an intraclass correlation coefficient above 0.89 for all tests. Steenholdt et al. (18) compared infliximab drug levels obtained using four different types of assays including ELISA, HMSA, radioimmunoassay (RIA) and functional cell-based reporter gene assay (RGA); however, significant disagreement existed between infliximab concentrations obtained between ELISA and HMSA(Prometheus), with a mean difference of 0.64 (0.15 to 1.12) μg/mL. Bodini et al. (19) evaluated an ELISA and HMSA (Prometheus) assay for adalimumab. Their data revealed a good correlation between the two methods with an *r* of 0.691 (*p* = 0.0003), however, they noted adalimumab concentrations measured by HMSA were consistently higher compared to those measured using ELISA.

The only emerging data on UST TDM was performed by Marini et al (21) who compared Janssen R&D ECLIA assay used in the pivotal clinical trials (9) with KU Leuven ELISA assay. They reported a strong agreement between those two assays. In our study, we aimed to compare the UST drug concentration level obtained using three commercially available assays, including two ELISA assays and one HMSA assay. A good correlation was observed for UST drug concentrations across the three assays and a good agreement was observed between the two ELISA tests, namely Dynacare and Theradiag. However, agreement was poor between the HMSA (Prometheus) and both ELISA tests as a result of an almost twofold increased difference in the absolute UST drug concentrations between the HMSA and both ELISA tests. We were unable to perform any qualitative analysis such as Fleiss Kapa test due to the fact that there are no clear cut-offs available in the current UST literature.

Our study highlights the potential limitations of extrapolation of absolute concentrations between assays using different techniques, particularly between HMSA and ELISA tests. In the absence of standardization of assays for TDM of UST, clinicians should be aware of the substantial absolute differences in UST drug concentrations between assays and ensure that interpretation of the results is based on the assay being used. Moreover, clinicians should avoid using different assays interchangeably, as this could lead to inappropriate management based on the differing results.
